# Embryonic Carcinoma Cells Show Specific Dielectric Resistance Profiles during Induced Differentiation

**DOI:** 10.1371/journal.pone.0059895

**Published:** 2013-03-22

**Authors:** Simin Öz, Christian Maercker, Achim Breiling

**Affiliations:** 1 Division of Epigenetics, DKFZ-ZMBH Alliance, German Cancer Research Center, Heidelberg, Germany; 2 Mannheim University of Applied Sciences, Mannheim, Germany; 3 Genomics and Proteomics Core Facilities, German Cancer Research Center, Heidelberg, Germany; Baylor College of Medicine, United States of America

## Abstract

Induction of differentiation in cancer stem cells by drug treatment represents an important approach for cancer therapy. The understanding of the mechanisms that regulate such a forced exit from malignant pluripotency is fundamental to enhance our knowledge of tumour stability. Certain nucleoside analogues, such as 2′-deoxy-5-azacytidine and 1β-arabinofuranosylcytosine, can induce the differentiation of the embryonic cancer stem cell line NTERA 2 D1 (NT2). Such induced differentiation is associated with drug-dependent DNA-damage, cellular stress and the proteolytic depletion of stem cell factors. In order to further elucidate the mode of action of these nucleoside drugs, we monitored differentiation-specific changes of the dielectric properties of growing NT2 cultures using electric cell-substrate impedance sensing (ECIS). We measured resistance values of untreated and retinoic acid treated NT2 cells in real-time and compared their impedance profiles to those of cell populations triggered to differentiate with several established substances, including nucleoside drugs. Here we show that treatment with retinoic acid and differentiation-inducing drugs can trigger specific, concentration-dependent changes in dielectric resistance of NT2 cultures, which can be observed as early as 24 hours after treatment. Further, low concentrations of nucleoside drugs induce differentiation-dependent impedance values comparable to those obtained after retinoic acid treatment, whereas higher concentrations induce proliferation defects. Finally, we show that impedance profiles of substance-induced NT2 cells and those triggered to differentiate by depletion of the stem cell factor OCT4 are very similar, suggesting that reduction of OCT4 levels has a dominant function for differentiation induced by nucleoside drugs and retinoic acid. The data presented show that NT2 cells have specific dielectric properties, which allow the early identification of differentiating cultures and real-time label-free monitoring of differentiation processes. This work might provide a basis for further analyses of drug candidates for differentiation therapy of cancers.

## Introduction

The induction of differentiation by treatment with natural ligands and synthetic drugs represents an important approach for cancer therapy [1], [2]. Tumours are thought to originate from cells with stem cell characteristics that have acquired aberrant gene expression patterns, mostly due to genetic and/or epigenetic mutations, which destabilise the homeostasis of cellular proliferation and differentiation [1], [3]. Cancer is thus characterised by a block in differentiation and by the induction of uncontrolled proliferation [3]. The identification and characterisation of substances that induce differentiation in human cancer cells therefore represents an important aspect in the development of novel cancer therapies.

A prominent example for a differentiation inducing drug is 2′-deoxy-5-azacytidine (decitabine, DAC), that has been suggested to induce differentiation by DNA demethylation [4]. A compound closely related to decitabine, 1β-arabinofuranosylcytosine (cytarabine, araC), induces differentiation without inhibiting DNA methylation [5]. DAC, araC and the structurally related drug 5-azacytidine (AZA), are used for the treatment of myeloid leukaemias, a group of diseases that is characterised by a differentiation block of precursor cells [6], [7]. While the precise molecular modes of action of these drugs are still not well understood, nucleoside analogues can be incorporated into DNA and thereby trigger DNA damage or other stress response pathways [8]. Indeed, we have recently shown that both DAC and araC induce neuronal differentiation in the embryonal carcinoma (EC) cell line NTERA2 D1 (NT2) by triggering degradation of OCT4 and other stem cell proteins via DNA damage pathways [9].

NT2 EC cells express high levels of stem cell specific transcription factors (especially OCT4 and NANOG), Polycomb Group (PcG) proteins and DNA methyltransferases. The cells also show significant levels of non-CpG methylation, a DNA mark restricted to pluripotent cells that is strongly reduced upon differentiation induction with all-trans-retinoic acid (RA), a conserved intercellular signaling molecule found in most vertebrates [10]. NT2 cells have not only been shown to differentiate along the neuronal lineage, but also show mesodermal and ectodermal lineage potential and thus represent a valuable human cancer stem cell model system [11], [12]. Cultures exposed to differentiation-inducing substances are usually rather heterogeneous and show a mixture of neuronal, ectodermal and mesodermal features [11]–[14]. Induction of differentiation with the natural ligand retinoic acid results in visible morphological changes only after prolonged treatment of at least three days [9], [15]. Changes in marker gene expression are even more delayed. Efficient reduction of stem cell factors or induced expression of neuronal markers becomes apparent only after several days of RA treatment [9], [13], [15]. In order to screen drug libraries for differentiation-inducing substances a fast method for early-identification of cellular differentiation is thus desirable.

Electrical cell-substrate impedance sensing (ECIS) is a label-free, non-invasive monitoring technique to study the formation of cell-matrix as well as cell-cell contacts during cell proliferation, cell migration, metastasis, wound healing, cellular differentiation and cancer development [16]–[18]. The method is based on the phenomenon that living cells behave as dielectric particles and thus alter the electrode impedance after attachment to a microelectrode surface. Impedance measurements at the electrode-cell interface are influenced by increasing cell number, increased adhesion, morphological changes and cell spreading [19]. We have previously used this non-invasive assay to measure impedance profiles of differentiating mesenchymal stem cells [20]. Mesenchymal stem cells (MSCs) induced for adipogenesis or osteogenesis *in vitro*, showed characteristic changes in dielectric properties, that were already visible within 24 hours. ECIS is thus a reliable tool for real-time monitoring of stem cell differentiation [20], [21].

To study immediate effects on impedance values, we analysed the onset of drug-induced differentiation in NT2 cells by ECIS. Already after 20 hours of retinoic acid induction we found a significant increase of impedance values. The slope/time ratios of the dielectric resistance profiles positively correlated with the employed concentration of RA. Further experiments determined the concentrations of nucleoside drugs that induced impedance changes with slope/time ratios comparable to those obtained with retinoic acid. These differentiation-specific effects could be separated from cytotoxicity. Finally, we show that differentiation induction by nucleoside drugs and retinoic acid is mainly caused by the reduction of the levels of stemness factors, in particular OCT4. Taken together, our work provides a basis for further real-time studies in living cells evaluating drug candidates as differentiation inducing agents for cancer therapy.

## Materials and Methods

### Cell Culture and Drug Treatment

The human cell line NT2 D1 [22], [23] was a kind gift from Peter W. Andrews (University of Sheffield). Cell line authentication was provided by LGC Standards (Teddington, report tracking no. 71008933). Cells were maintained in Dulbecco’s Modified Eagle Medium (DMEM) supplemented with 10% FCS (Invitrogen), 200 U/ml penicillin (Gibco) and 200 µg/ml streptomycin (Gibco). NT2 cells were induced to differentiate (if not mentioned otherwise) with 10 µM all-trans retinoic acid (Sigma), 1 µM deoxycytidine (Sigma), 1 µM azacytidine (Sigma), 1 µM decitabine (Sigma), 1 µM cytarabine (Sigma), 5 mM hexamethylene bisacetamide (Sigma) and 50 µM fibroblast growth factor 2 (Novitec) in 5% CO_2_ at 37°C.

### RNA Isolation and qRT PCR

Total RNA was isolated from NT2 D1 cells using the Trizol reagent (Invitrogen) or the RNeasy kit (Qiagen), following the manufacturer’s recommendations. Total RNA (500 ng) was reverse transcribed using Superscript III (Invitrogen). Quantitative RT-PCR was performed utilising the LightCycler 480 System (Roche). 1 µl of cDNA was used for 10 µl PCR reaction using Absolute QPCR SYBR Green Mix (Thermo Scientific) under following conditions: 1 cycle at 95°C for 15 min followed by 50 cycles at 95°C for 15 s, at 60°C for 40 s. All samples were measured in triplicates. Cycle threshold numbers for each amplification were measured with the LightCycler 480 software, and relative expression values were calculated and normalised using *β-actin* as an internal standard. For RT-primer sequences see Table S5.

### Impedance Measurements

NT2 D1 cells (2×10^4^ per well) were seeded in triplicates or quadruplicates and grown in 400 µl DMEM supplemented with 10% FCS (Invitrogen), 200 U/ml penicillin (Gibco) and 200 µg/ml streptomycin (Gibco) on 8W10E+ ECIS Cultureware arrays (Applied Biophysics) that contain 40 250-µm gold electrodes per well. Cells were then treated as indicated. Measurements were carried out in cell culture medium (without medium changes) and the arrays were kept in the incubator with 5% CO_2_ at 37°C. The arrays were measured on an ECIS™ Model 1600 (Applied Biophysics) at 45 kHz in 5-minute intervals for 96 hours. Data were normalised to their starting values. Analysis was done on ECIS software based on the model developed by Giaever and Keese [19].

### Staining and Microscopy

At ECIS endpoints, cells attached to the arrays were washed with 1X Phosphate Buffered Saline (PBS) and fixed with 4% paraformaldehyde. Cells were permeabilised with 0.1% TritonX-100, stained with Phalloidin Tritc (Sigma) and DAPI (Invitrogen) in PBS for 1 hour and washed. After staining, 8-well chamber tops were removed from the base slides and mounting media and cover slips were added. Fluorescence images were taken on Zeiss Axioskop 2 Plus. Phase contrast images of living cells were taken on a Leica DM-IRBE inverse microscope.

### Protein Depletion via siRNAs

For the depletion of OCT4, specific ON-TARGETplus SMARTpool siRNAs (Dharmacon) were used as described [9]. In brief, NT2 cells were seeded in quadruplicate into 8W10E+ ECIS Cultureware arrays (Applied Biophysics) at a density of 2×10^4^ per well, with 400 µl of medium (see above). Cells were transfected with siRNAs using the DharmaFECT1 transfection reagent (Dharmacon) according to the manufacturer's instructions. The final siRNA concentration was 50 nM. Scrambled siRNAs for negative control experiments were also obtained from Dharmacon. Impedance measurements were started immediately after transfection, as described above and carried out during a 6 day period. Medium was changed once after 3 days. Impedance peaks caused by the medium change were equalised during data analysis with the ECIS software.

### Statistical Analysis and Bioinformatics

Two-tailed student’s t-test was used for statistical analysis of ECIS and qRT-PCR data. To determine the slope maxima of the differentiation-induced impedance data, we applied a cubic smoothing spline with 10 degrees of freedom to the resistance values and performed a generalised cross validation to the data [24]. The steepest rise in impedance was determined as the time point with the maximum slope.

## Results

### Monitoring RA-induced Differentiation using Electric Impedance Sensing

**Figure 1:**
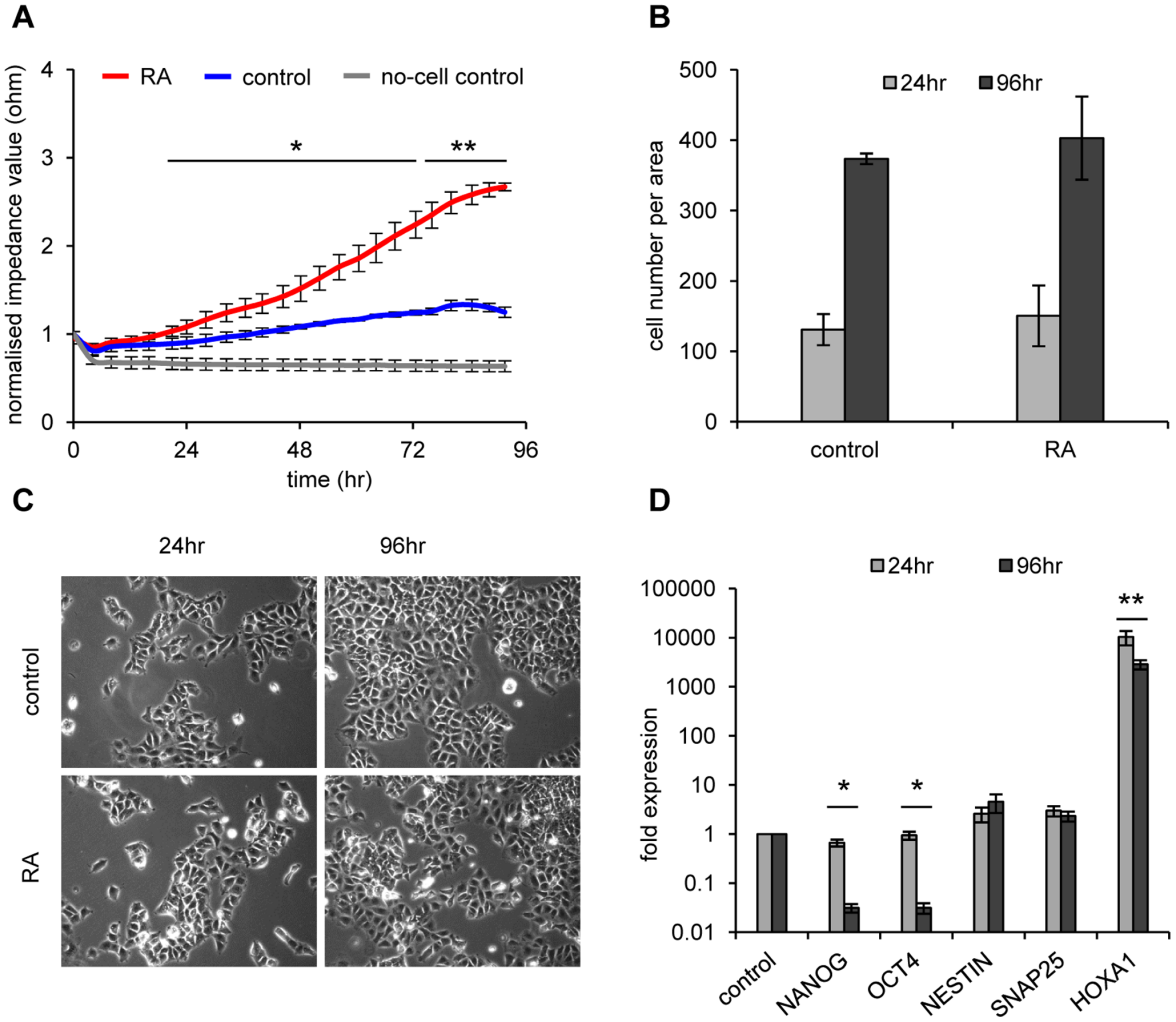
Retinoic acid induced neuronal differentiation of NT2 EC cells. (**A**) Impedance profiles comparing RA-induced (10 µM - red) and untreated NT2 cells (blue) during a 4 day period. The mean of three independent experiments is shown. Standard deviations are indicated by error bars every four hours. Measurements were executed at 45 kHz in 5-minute intervals for 96 hours. Normalised resistance values were compared by two-tailed Student’s t-test. After 20 hours of RA treatment differences in impedance values start to become statistically significant (*p<0.05, **p<0.005). Black lines show regions with significant differences in respect to the untreated cell control. (**B**) Average cell numbers of three replicates of untreated and RA-treated NT2 cells after 24 and 96 hours do not differ significantly. Standard deviations are indicated by error bars. (**C**) Microscopic images (10× magnification) of NT2 control cells and NT2 cells treated with RA for 24 and 96 hours. No clear differentiation phenotype becomes apparent for the RA treatment. (**D**) qRT-PCR expression analysis of stem cell factors *NANOG*, *OCT4* and the differentiation markers *NESTIN*, *SNAP25* and *HOXA1* in RA- treated and control cells after 24 and 96 hours of treatment. Data is shown in logarithmic scale. Only *HOXA1* is prominently induced by retinoic acid at both time points. The stemness genes are only found reduced after 96 hours of RA treatment. All qRT-PCR measurements were repeated at least three times and internally normalised to the corresponding *β-actin* values. Standard deviations are indicated by error bars. Two-tailed student’s t-test showed significant differences when comparing expression levels of *OCT4*, *NANOG* and *HOXA1* at 24 hours with the expression levels at 96 hours. (*p<0.05, **p<0.005).

RA-induced neuronal differentiation of NT2 cells [15], [25] is a comparably slow process that usually requires several weeks of treatment before morphological changes become visible, even if gene expression patterns change more rapidly [15]. In order to use a non-invasive method and to directly monitor differentiation induction *in vitro*, we seeded NT2 cells (2×10^4^ per well) into eight-well ECIS-arrays (8WE10+) and measured resistance changes at 45 kHz every 5-minutes in the absence or presence of RA over a four day period (96 hours). As shown in Figure 1A, untreated NT2 cells showed only a weak increase of frequency dependent impedance values over four days, which was most likely mainly caused by the increasing cell number. In striking contrast, treatment with 10 µM RA led to a significant increase of impedance values starting with 20 hours of treatment (Fig. 1A). Total cell numbers did not differ significantly between untreated and RA-treated cells at 24 or 96 hours of growth (Fig. 1B), indicating that impedance differences were not due to differing growth rates. Also, overall the morphology of both cell populations was very similar (Fig. 1C). Early onset of differentiation is usually monitored by marker gene expression using quantitative reverse transcription PCR (qRT-PCR) on total RNA isolated from growing cells. As shown in Fig. 1D, transcription of the stem cell factors *NANOG* and *OCT4* was significantly down-regulated in RA-treated cells, but only after 96 hours of RA treatment, whereas the specific differentiation markers *NESTIN*, *SNAP25* and *HOXA1* were induced. As expected, *HOXA1*, a very early and prominent marker of differentiation, showed a strong increase of expression within 24 hours (see also ref. 9). However, expression differences of the other genes investigated were barely visible by qRT-PCR within the first day after start of treatment (Fig. 1D, light grey bars). Thus, impedance measurement is a highly sensitive and robust method to follow the early onset of RA-induced differentiation.

**Figure 2:**
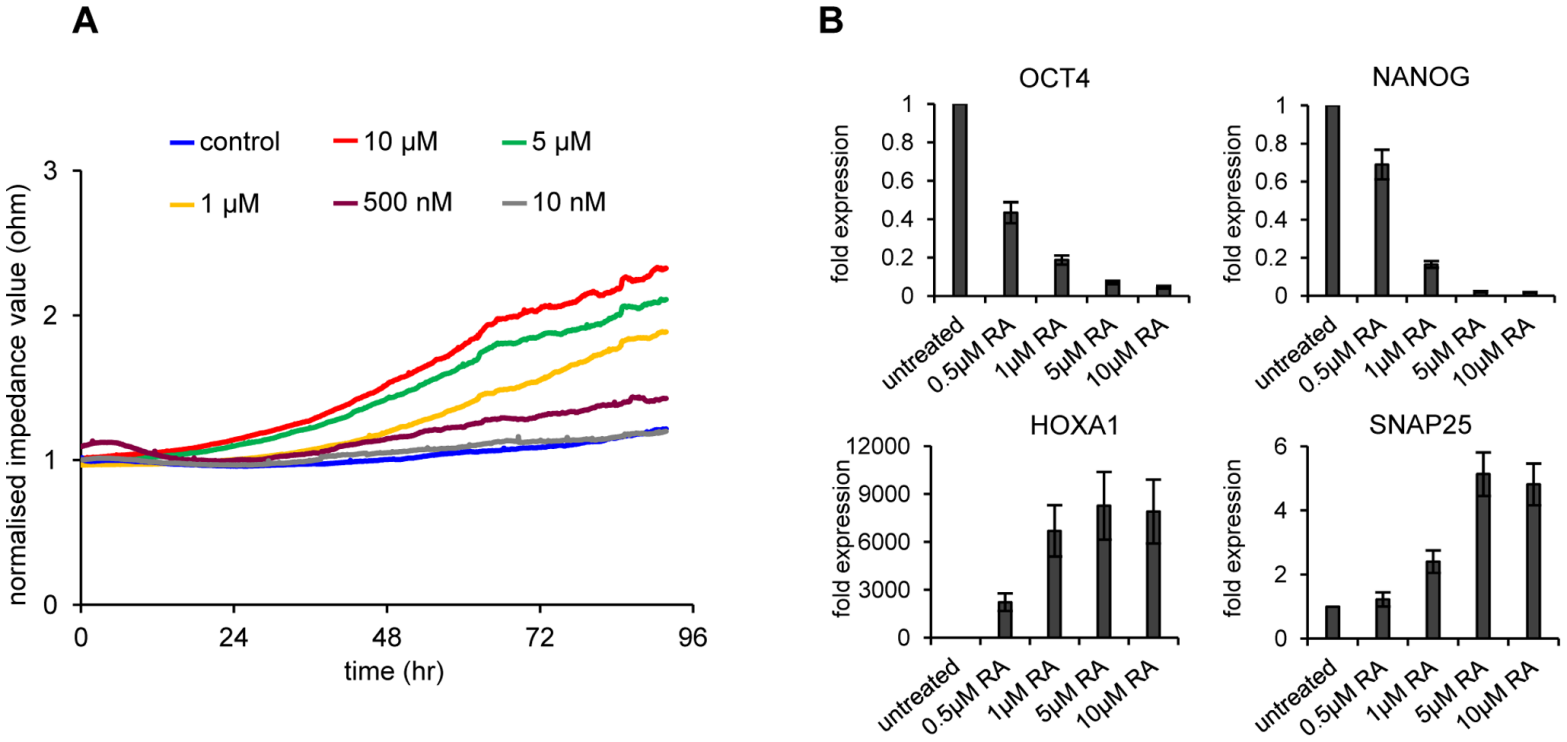
Induced concentration-dependent differentiation of NT2 cells by RA. (**A**) Impedance profiles comparing induction profiles of different RA concentrations during a 4 day period. Measurements were executed at 45 kHz in 5-minute intervals for 96 hours. The mean of three independent experiments is shown. Standard deviations are not shown to avoid crowding of the diagram. For single diagrams including standard deviations and statistical tests for these data sets see Fig. S1. (**B**) qRT-PCR expression analysis of stem cell factors *NANOG*, *OCT4* and the differentiation markers *HOXA1* and *SNAP25* and in RA- treated and control NT2 cells after 96 hours of treatment. The concentration of RA employed correlates negatively with the expression of stem cell factors, but positively with the expression of differentiation markers. All qRT-PCR measurements were repeated at least three times and internally normalised to the corresponding *β-actin* values. Standard deviations are indicated by error bars.

In order to monitor the effect of RA concentration on differentiation induction, we treated NT2 cells with different concentrations of retinoic acid and registered the dielectric resistance profiles (Fig. 2A, Fig. S1). Increasing RA concentrations lead to increased resistance (as indicated by the time point of first statistical significant difference in impedance values of control experiments versus RA treatment, see Fig. S1) and also a steeper slope of the impedance profiles. This correlated with the state of differentiation, as confirmed by the measurement of marker gene expression after 96 hours of treatment (Fig. 2B). We then chose two parameters that characterise RA-induced resistance changes: the slope of the curve obtained when joining the single points of resistance measurements and the time point when the maximum slope is reached. The higher the slope and the earlier the slope maximum, the faster and stronger are the resistance changes that reflect ongoing differentiation. We therefore analysed the dataset shown in Fig. 2A by applying a cubic smoothing spline and determined for each treatment the maximum slope and the time point the maximum was reached. Then, by calculating the slope/time ratio (in order to compensate for low, but early slope maxima), a clear positive correlation between RA concentration, marker gene expression and maximum slope and time was found (Table S1). Thus the slope/time ratio can be used as an early marker for differentiation.

### Electric Impedance Sensing of NT2 Cells Treated with a Panel of Differentiation Inducing Drugs

**Figure 3:**
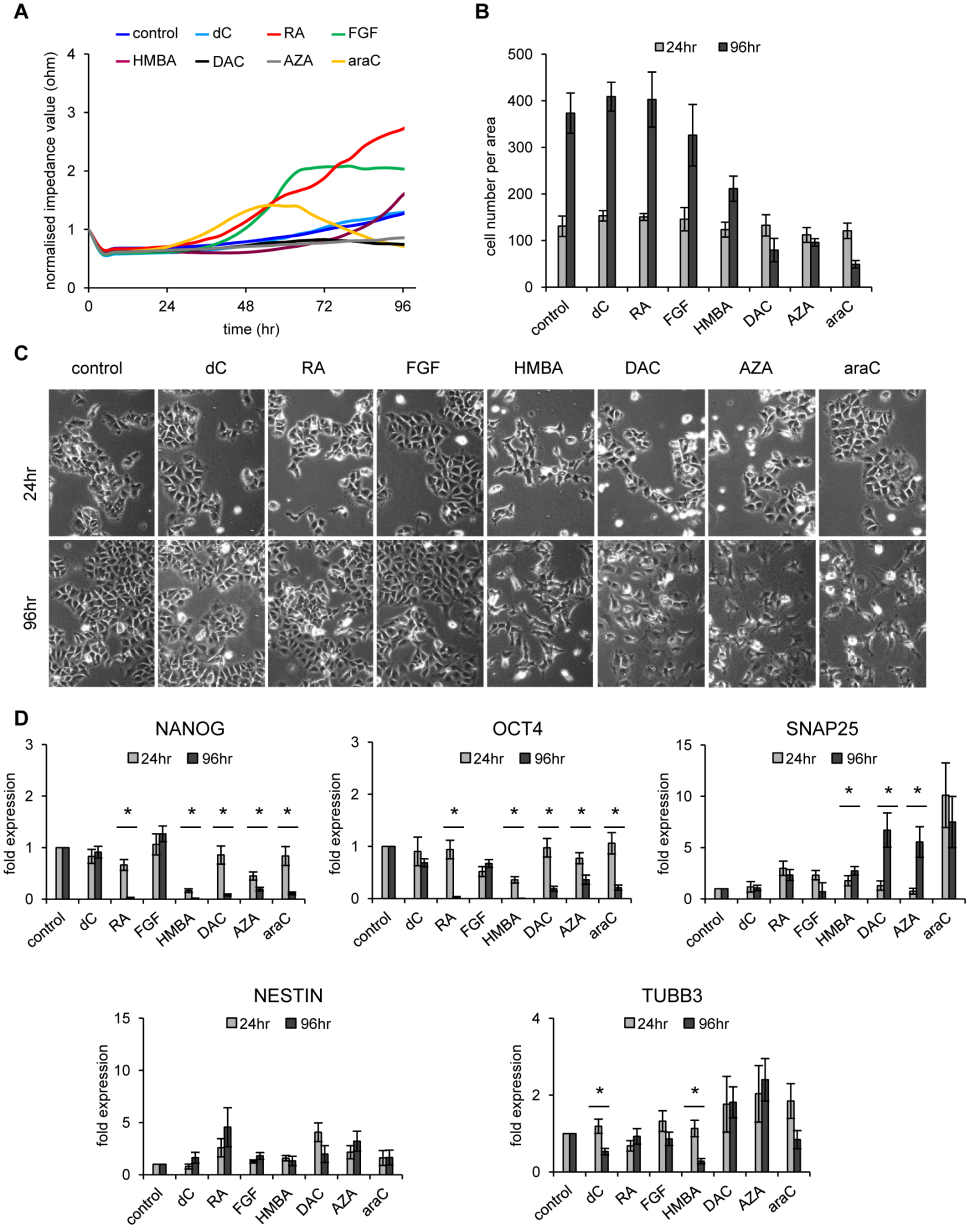
Induced differentiation by a defined panel of drugs. (**A**) Impedance profiles comparing NT2 cells treated with retinoic acid (RA, 10 µM), hexamethylene bisacetamide (HMBA, 5 mM), 5-azacytidine (AZA, 1 µM), deoxycytidine (dC, 1 µM), fibroblast growth factor 2 (FGF, 50 µM), 2′-deoxy-5-azacytidine (DAC, 1 µM) and 1β-arabinofuranosylcytosine (araC, 1 µM) during a 4 day period. Measurements were executed at 45 kHz in 5-minute intervals for 96 hours. The mean of three independent experiments is shown. Standard deviations are not shown to avoid crowding of the diagram. For single diagrams including standard deviations and statistical tests for these data sets see Fig. S2. (**B**) Average cell numbers of three replicates of untreated and treated NT2 cells after 24 and 96 hours. Nucleoside drugs are cytotoxic at the concentrations used and show significant growth inhibition. Standard deviations are indicated by error bars. (**C**) Microscopic images (10× magnification) of NT2 control cells and NT2 cells treated with the various substances mentioned in (A) after 24 and 96 hours of treatment. (**D**) qRT-PCR expression analysis of stem cell factors *NANOG*, *OCT4* and the differentiation markers *NESTIN*, *SNAP25* and *TUBB3* in treated and NT2 control cells after 24 and 96 hours of treatment. All qRT-PCR measurements were repeated at least three times and internally normalised to the corresponding *β-actin* values. Standard deviations are indicated by error bars. Treatments showing significant differences comparing expression levels at 24 hours with those at 96 hours are marked with an asterisk (two-tailed student’s t-test; p<0.05).

In order to expand our analyses to other differentiation-inducing substances and to monitor NT2 cells induced to differentiate into other lineages, we treated the cells with the nucleoside analogues araC, DAC and AZA and measured the impedance profiles (Fig. 3A, Fig. S2). In parallel experiments, differentiation was induced by Fibroblast Growth Factor 2 (FGF2; bFGF) and hexamethylene bisacetamide (HMBA). Expression of bFGF increases during retinoic acid induced differentiation of NT2 cells and bFGF treatment of floating spheres of NT2 cells has been shown to trigger terminal differentiation into neurons [14], [25]–[27]. In addition, mesodermal features in aggregated NT2 cells after prolonged treatment with bFGF have also been reported [12]. HMBA treatment of NT2 cells has been shown to result in the expression of marker genes usually associated with epithelial structures, suggesting that HMBA triggers differentiation into epidermal ectoderm [11], [13].

As shown in Figure 3A (see also Fig. S2), araC treatment led to an early increase of resistance, indicating onset of differentiation. This effect was followed by a significant drop below the values of the deoxycytidine (dC) control (Fig. 3A, Fig. S2F). All three nucleoside drugs induced strong proliferation defects after 24 hours of growth (Fig. 3B), most likely by triggering DNA-damage dependent apoptotic pathways [9], which led to low cell numbers and reduced impedance values.

bFGF induced an increase in resistance after 48 hours of treatment (Fig. 3A, Fig. S2B). Cells treated with HMBA, however, showed values in the range of the untreated control. HMBA treatment also reduced the cell number in the culture, but in contrast to araC, an increase in resistance was observed after 72 hours (Fig. 3A, Fig. S2C).

The analysis of the slope/time ratios reveals similar values for RA and HMBA, reflecting a similar potential to induce differentiation (Table S2). Nevertheless, after HMBA treatment the maximum slope was reached much later, indicating a distinct differentiation pathway. bFGF and araC show higher slope/time ratios, due to an earlier onset of increasing resistance for araC and a steeper slope for bFGF, again reflecting different modes of differentiation induction. Taken together, RA, HMBA, bFGF and araC showed significant induction potentials, resulting in specific dielectric resistance profiles and slope/time ratios, whereas AZA and DAC treated cells had a similar profile as the controls after 24 hours of treatment. Nevertheless, for araC, DAC and AZA the strong reduction of surviving cells lead to declining impedance values at later time points (Fig. 3A, Fig. 3B, Fig. S2D, S2E and S2F).

As already observed for retinoic acid (Fig. 1C), the majority of differentiation-inducing factors did not induce any significant morphological differences after 24 hours of treatment (Fig. 3C). However, HMBA treated cells, which had shown a delayed increase in dielectric resistance, appeared morphologically different already after 24 hours of treatment (Fig. 3C). After 96 hours of incubation, onset of differentiation was visible for all differentiation-inducing factors (Fig. 3C). In addition, araC, DAC and AZA treated cells showed clear reduction of cell numbers due to the cytotoxicity of these compounds.

As shown by qRT-PCR in Figure 3D, stem cell factors (*OCT4* and *NANOG*) were less expressed after 96 hours of treatment with all substances (except bFGF), indicating ongoing differentiation and loss of pluripotency. Consistent with the morphological alterations, expression of both genes was only weakly reduced after 24 hours. Furthermore, differentiation markers (*SNAP25*, *NESTIN*, *TUBB3*) were only moderately increased after 96 hours of treatment (Fig. 3D). At 24 hours, PCR-based expression analysis of differentiation marker genes as well as phase contrast microscopy failed to clearly indicate onset of differentiation (Fig. 3C, 3D, light grey bars). However, the drug-specific impedance values suggest that differentiation already starts within the first day of treatment, especially with RA, bFGF and araC (Fig. 3A, Fig. S2). Thus treatment-induced early differentiation steps obviously trigger changes in cell-extracellular matrix contacts, leading to increased resistance (Fig. 1A, Fig. 2A, Fig. 3A, Table S2). This finding underscores the value of ECIS analysis, especially to analyse early differentiation states, and is also in accordance with recent *in vitro* differentiation data of MSCs, which revealed changes in impedance profiles already within the first hours of adipogenic or osteogenic differentiation [20], [21]. Our data further suggest that lineage specific morphological changes influence impedance values in different ways, leading to characteristic resistance profiles and slope/time ratios.

### Concentration Dependence of Drug Induced Impedance Curves

**Figure 4:**
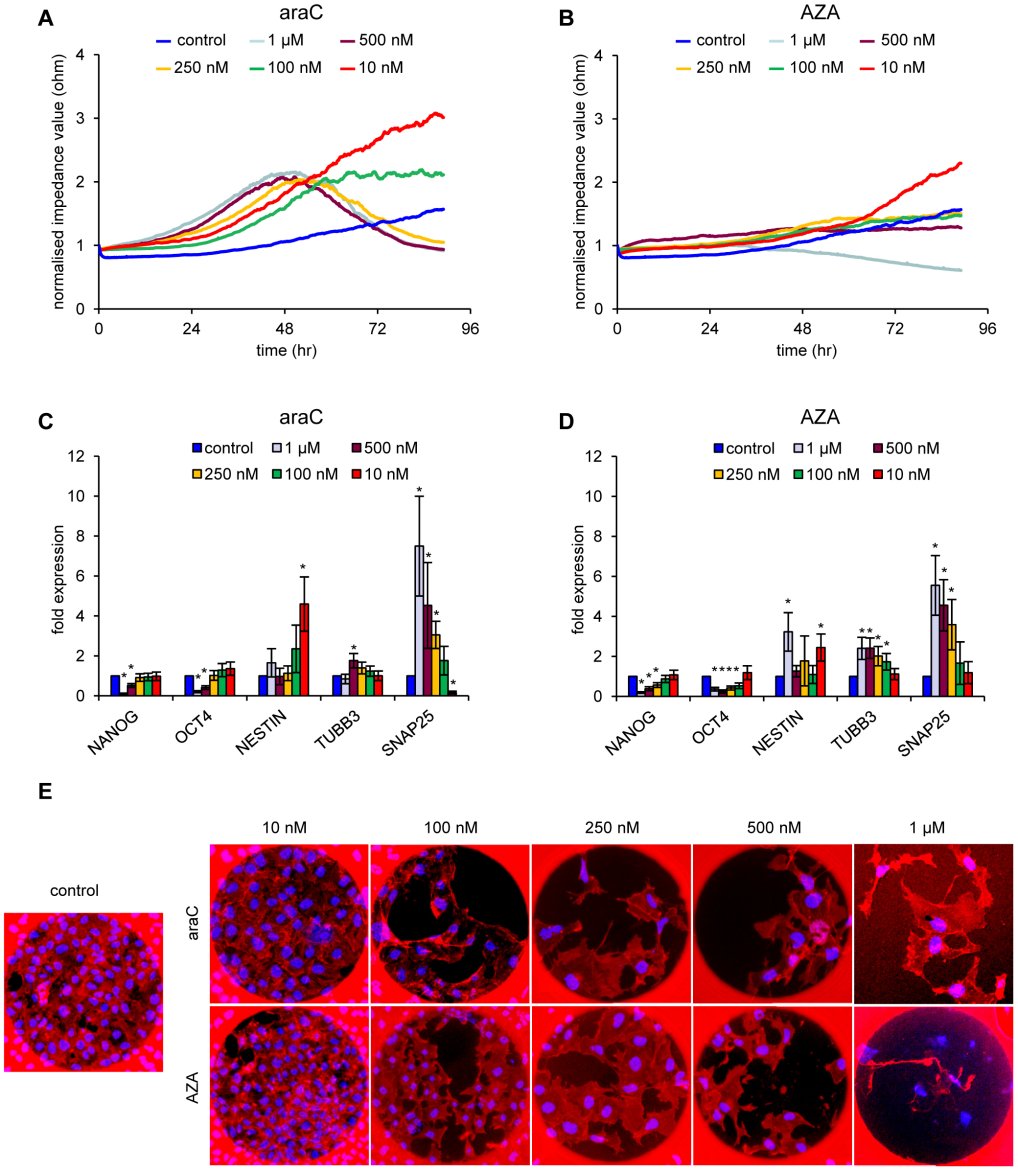
Induced concentration-dependent differentiation by araC and AZA. (**A**) Impedance profiles of NT2 cells treated with different concentrations of 1β-arabinofuranosylcytosine (araC) during a 4 day period. Concentrations above 100 nM are severely cytotoxic, which leads to a drastic drop in impedance values after 48 hours. Measurements were executed at 45 kHz in 5-minute intervals for 96 hours. One representative experiment is shown. For single diagrams showing the mean of at least three experiments including standard deviations and statistical tests see Fig. S3A. (**B**) Impedance profiles of NT2 cells treated with different concentrations of 5-azacytidine (AZA) during a 4 day period. Concentrations above 100 nM strongly induce proliferative defects, which prevents the increase of impedance values. Measurements were executed at 45 kHz in 5-minute intervals for 96 hours. One representative experiment is shown. For single diagrams showing the mean of at least three experiments including standard deviations and statistical tests see Fig. S3B. (**C**) qRT-PCR expression analysis of stem cell factors *NANO*G, *OCT4* and the neuronal differentiation markers *NESTIN*, *SNAP25* and *TUBB3* in NT2 cells treated with different concentrations of araC and control cells after 96 hours of treatment. All qRT-PCR measurements were repeated at least three times and internally normalised to the corresponding *β-actin* values. Standard deviations are indicated by error bars. Expression levels of the respective genes showing significant differences compared with the untreated control are marked an asterisk (two-tailed student’s t-test; p<0.05). (**D**) qRT-PCR expression analysis of stem cell factors *NANO*G, *OCT4* and the neuronal differentiation markers *NESTIN*, *SNAP25* and *TUBB3* in NT2 cells treated with different concentrations of AZA and control cells after 96 hours of treatment. All qRT-PCR measurements were repeated at least three times and internally normalised to the corresponding *β-actin* values. Standard deviations are indicated by error bars. Expression levels of the respective genes showing significant differences compared with the untreated control are marked an asterisk (two-tailed student’s t-test; p<0.05). (**E**) Phalloidin staining of growing cultures. Flourescence images (10× magnification) of NT2 control cells and NT2 cells treated with the indicated concentrations of AZA and araC. The circular dark region is the electrode measuring area covered by the cells. Cells were stained with Phalloidin TRITC (red) and DAPI (blue).

As shown in Figures 3A and 3B, the nucleoside drugs were cytotoxic at the concentrations used initially (1 µM), leading to reduced cell numbers after prolonged treatment, therefore impeding the monitoring of differentiation-dependent impedance changes. We addressed the possibility to separate their influence on differentiation induction from cytotoxic side effects by lowering the concentration of these compounds. As shown in Figs. 4 and S3, 10 nM araC induced impedance values that were similar to RA-treated cells, without triggering cell death, with a slope/time ratio comparable to the one obtained with RA at 10 µM (Table S2 and Table S3). Up to 48 hours, also araC concentrations higher than 10 nM induced increased dielectric resistance, leading to positively correlated slope maxima (Table S3). However, at later time points cytotoxicity lead to significantly reduced growth and a drop in impedance (Fig. 4A, Fig. S3). Treatment with AZA at 10 nM lead to a similar, but retarded increase in resistance, again comparable to RA at 10 µM, which is also reflected by the slope/time ratio (Fig. 4B; Fig. S3, Table S3). Thus, as with araC, 10 nM AZA triggered differentiation of NT2 cells without inducing proliferation defects. Higher concentrations of AZA were mostly toxic for the cells.

End point qPCR of stem cell and differentiation markers showed only moderate changes for low concentrations (100 nM and 10 nM) of araC and AZA (Fig. 4C and 4D). Only *NESTIN* expression was significantly increased in araC-treated cells. It should also be noted that the moderate differentiation phenotypes obtained with 10 nM araC or AZA were morphologically similar to those of RA-induced cultures (see Fig. 1C). Stronger differentiation phenotypes and gene expression changes were only obtained with higher, cytotoxic drug concentrations (Fig. 4C–E). These results further underscore the sensitivity of the ECIS method in detecting early onset of differentiation.

### Electric Impedance Sensing of OCT4-depleted NT2 Cells

**Figure 5:**
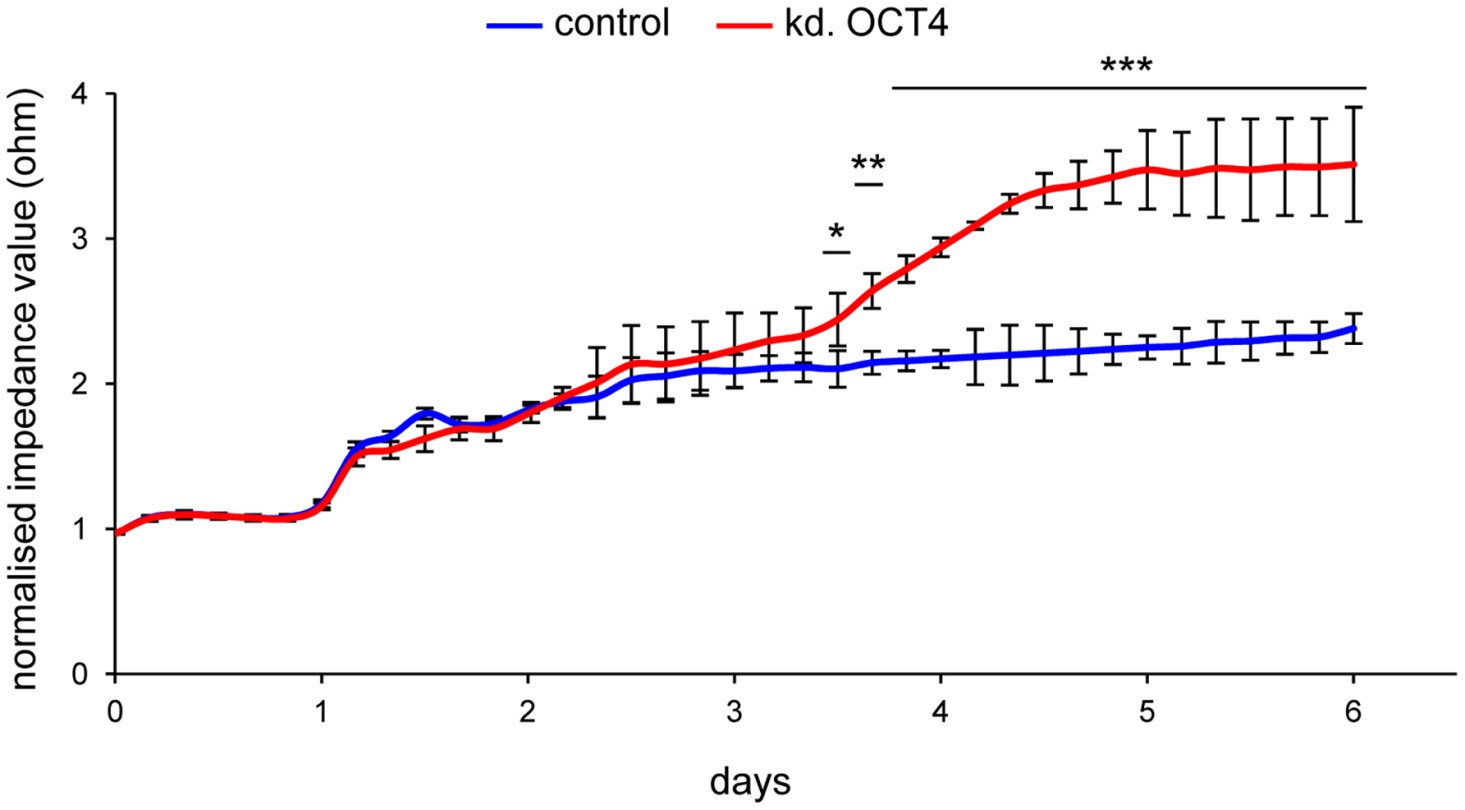
Induced differentiation by RNAi-mediated depletion of OCT4. Impedance profiles of control NT2 cells (blue - scrambled knock down) and NT2 cells depleted for OCT4 (red) during a 6 day period. Measurements were executed at 45 kHz in 5-minute intervals for 6 days. The mean of three independent experiments is shown. Standard deviations are indicated by error bars every four hours. Student’s t-test was used for statistical analysis. Differences between control and knock down experiments in the indicated regions have been found to be statistically significant (*p<0.05, **p<0.005, ***p<0.001).

We have recently shown that siRNA-mediated depletion of the stem cell specific protein OCT4 induces neuronal differentiation in NT2 cells [9]. In order to analyse if reduction of OCT4 levels alone will lead to an increase of impedance levels in a similar way as retinoic acid or drug treatment, we seeded NT2 cells into ECIS-arrays and depleted OCT4 by siRNA transfection, using conditions that cause more than 90% reduction of *OCT4* mRNA levels [9]. As shown in Figure 5 increased resistance became apparent for the OCT4 depleted population after 2–3 days, reaching levels comparable to RA treatment around day 4. The delay of 2–3 days is caused by the knock down procedure, as efficient turnover of OCT4 protein is only achieved after 3 days [9], which also explains the observed increase in impedance over this period, as the cells continued to grow (Fig. 5). After substantial depletion of OCT4 was achieved at day 3, the cells started to differentiate, leading to a subsequent increase in resistance values (Fig. 5). Interestingly, when calculating the slope/time ratio (Table S4), we observed very similar values to the ones found in RA treated cells (compare Tables S1, S2 and S4). These findings show that differentiation induction of NT2 cells is triggered by the reduction of OCT4 and provide important confirmation for the argument that the observed changes in resistance were indeed caused by the onset of cellular differentiation.

## Discussion

In this study we demonstrate that treatment with well characterised differentiation triggering substances induces distinct dielectric changes in differentiating NT2 cell populations. We were able to generate impedance profiles during the first days of differentiation that allow to monitor the onset of differentiation very early (after 20 hours), when other phenotypic changes or differentiation specific marker gene expression patterns are not yet apparent. Further, by calculating slope/time ratios of each data set, we obtained a measure for the degree of induced differentiation. Impedance analysis also seems to allow the correlation of lineage choices with specific resistance profiles that could enable the prediction of differentiation pathways induced by specific drugs (RA, bFGF - early max. slope) from epidermal differentiation (HMBA - late max. slope).

MSCs show specific impedance profiles during adipogenic or osteogenic differentiation, caused by the modulation of cellular contacts with the gold electrodes or the extracellular matrix [20], [21]. As shown in this work, RA-induced neuronal differentiation of NT2 cells is also accompanied by specific interactions with the extracellular matrix (ECM) that change during later stages. Untreated NT2 cells express *alpha5beta1 integrin*, which specifically interacts with fibronectin, whereas NT2 neuron-like cells express *alpha3beta1 integrin* with LAMIN-5 as a ligand in the extracellular matrix [28]. During epithelial transition induced by HMBA, NT2 cells also show specific morphological characteristics. The nuclear to cytoplasm ratio is increased and the actin cytoskeleton is reorganised [29]. Impedance sensing thus seems to discriminate between these different modes of interaction with the extracellular matrix, which are specific for each differentiation pathway. Extracellular matrix molecules can also directly influence the efficiency of differentiation *in vitro* [20]. Besides cell-ECM interactions, also cell-cell contacts are of outmost importance during differentiation. Changes in cell membrane capacitance also can be described by an ECIS scan with different frequencies [21]. For example, connexins, expressed during neuronal differentiation [28] or desmosomes, expressed during epithelial differentiation [29] might come into focus in this respect.

Further we show that the previously described drug-induced differentiation using nucleoside analogues [9] induces similar impedance profiles and slope/time ratios as the natural ligand retinoic acid. The activation of differentiation and the maintenance of differentiation-specific gene expression patterns require substantial epigenetic modulation, especially changes in PcG presence, histone modification patterns and also DNA methylation [30]–[33]. We have previously used the DNMT inhibitor 2′-deoxy-5-azacytidine to induce hypomethylation and differentiation in NT2 cells [9]. Nevertheless, we observed even stronger differentiation induction by cytarabine, a drug that has no epigenetic modulatory potential and does not trigger any changes in DNA methylation [9]. This suggested a mechanism of drug-dependent differentiation that does not interfere directly with the epigenetic maintenance system.

RA- and nucleoside-drug induced NT2 cells showed very similar early impedance profiles. At higher concentrations of the drugs, cytotoxicity became predominant, resulting in reduced impedance. The ECIS assay allowed to determine the concentration thresholds of the tested drugs that were sufficient for the induction of differentiation without triggering cytotoxic side effects. At concentrations as low as 10 nM, araC and AZA induced differentiation-specific impedance profiles very similar to RA treatment that were stably increasing over more than three days. At concentrations above 100 nM, cytotoxicity became prominent, although surviving cells showed strong differentiation phenotypes. For both DAC and AZA, dose dependent dual mechanisms have been described, with cytotoxic and anti-proliferative effects at high doses and DNA hypomethylation at low doses [34]. Our impedance data further support this concept and may indicate that low doses of these drugs, including araC, can specifically induce differentiation in cancer stem cell populations.

Depletion of OCT4 by RNA interference induced similar resistance profiles and slope/time ratios as RA and low concentrated nucleoside drugs. This confirms the hypothesis that induced differentiation is caused predominantly by the reduction of OCT4 levels, either by proteolytic degradation (as for the nucleoside drugs) or by the transcriptional down-regulation of the gene (as for the natural ligand retinoic acid) [9]. Since cell membrane capacitance obviously is an early marker of differentiation, lower (1 kHz to 8 kHz) and higher frequencies (62.5–64 kHz) to measure the multifrequency complex impedance (Z*) might improve the characterisation of the drug induced cell inherent dielectric properties [21]. We so far only monitored impedance at 45 kHz. Measurements at other frequencies possibly will help to describe cell-cell vs. cell-matrix interactions in more detail. Nevertheless, our work shows that impedance sensing is a robust and sensitive method to describe the effects of differentiation inducing drugs by measuring the dielectric properties of cells in real-time. Using this method, very early differentiation processes could be followed within the first day after drug treatment in non-invasive conditions. Therefore, our work can serve as a basis for a more detailed analysis of molecular effects of signalling pathways involved in cellular differentiation and for the screening for drugs that modulate cellular phenotypes.

## Supporting Information

Figure S1 **Induced concentration-dependent differentiation by RA.** Impedance profiles comparing untreated NT2 with cells treated with 10 nM RA (**A**), 500 nM RA (**B**), 1 µM RA (**C**), 5 µM RA (**D**) and 10 µM RA (**E**) are shown. Measurements were executed at 45 kHz in 5-minute intervals for 96 hours. Each experiment was repeated at least three times. Standard deviations are indicated by error bars every four hours. Student’s t-test was used for statistical analysis (*p<0.05. **p<0.005). Black lines show regions with significant differences in respect to the untreated control.
(TIF)

Figure S2 **Induced differentiation by a panel of drugs.** Impedance profiles comparing untreated NT2 with cells treated with 1 µM dC (**A**), 50 µM bFGF (**B**), 5 mM HMBA (**C**), 1 µM DAC (**D**), 1 µM AZA (**E**) and 1 µM araC (**F**) are shown. Measurements were executed at 45 kHz in 5-minute intervals for 96 hours. Each experiment was repeated at least three times. Standard deviations are indicated by error bars every four hours. Student’s t-test was used for statistical analysis (*p<0.05. **p<0.005). Black lines show regions with significant differences in respect to the dC control.
(TIF)

Figure S3 **Induced concentration-dependent differentiation by araC and AZA.** (**A**) Impedance profiles comparing untreated NT2 cells (dark blue) and cells treated with 1 µM (light blue), 500 nM (purple), 250 nM (yellow), 100 nM (green) and 10 nM (red) araC. (**B**) Impedance profiles comparing untreated NT2 cells (dark blue) and cells treated with 1 µM (light blue), 500 nM (purple), 250 nM (yellow), 100 nM (green) and 10 nM (red) AZA. Measurements were executed at 45 kHz in 5-minute intervals for 96 hours. Each experiment was repeated at least three times. Standard deviations are indicated by error bars every four hours. Student’s t-test was used for statistical analysis (*p<0.05. **p<0.005). Black lines show regions with significant differences in respect to the control.
(TIF)

Table S1 **Slope maxima of RA-treated NT2 cells.**
(PDF)

Table S2 **Slope maxima of drug-treated NT2 cells.**
(PDF)

Table S3 **Slope maxima of araC- and AZA-treated NT2 cells.**
(PDF)

Table S4 **Slope maxima of OCT4-depleted NT2 cells.**
(PDF)

Table S5 **RT-Primer pairs used in this study.**
(PDF)
