# Supplementary figures and images for: Embryonic Carcinoma Cells Show Specific Dielectric Resistance Profiles during Induced Differentiation
Source: PLoS One. 2013 Mar 22;8(3):e59895. doi: 10.1371/journal.pone.0059895 (PMC3606267; doi:10.1371/journal.pone.0059895)

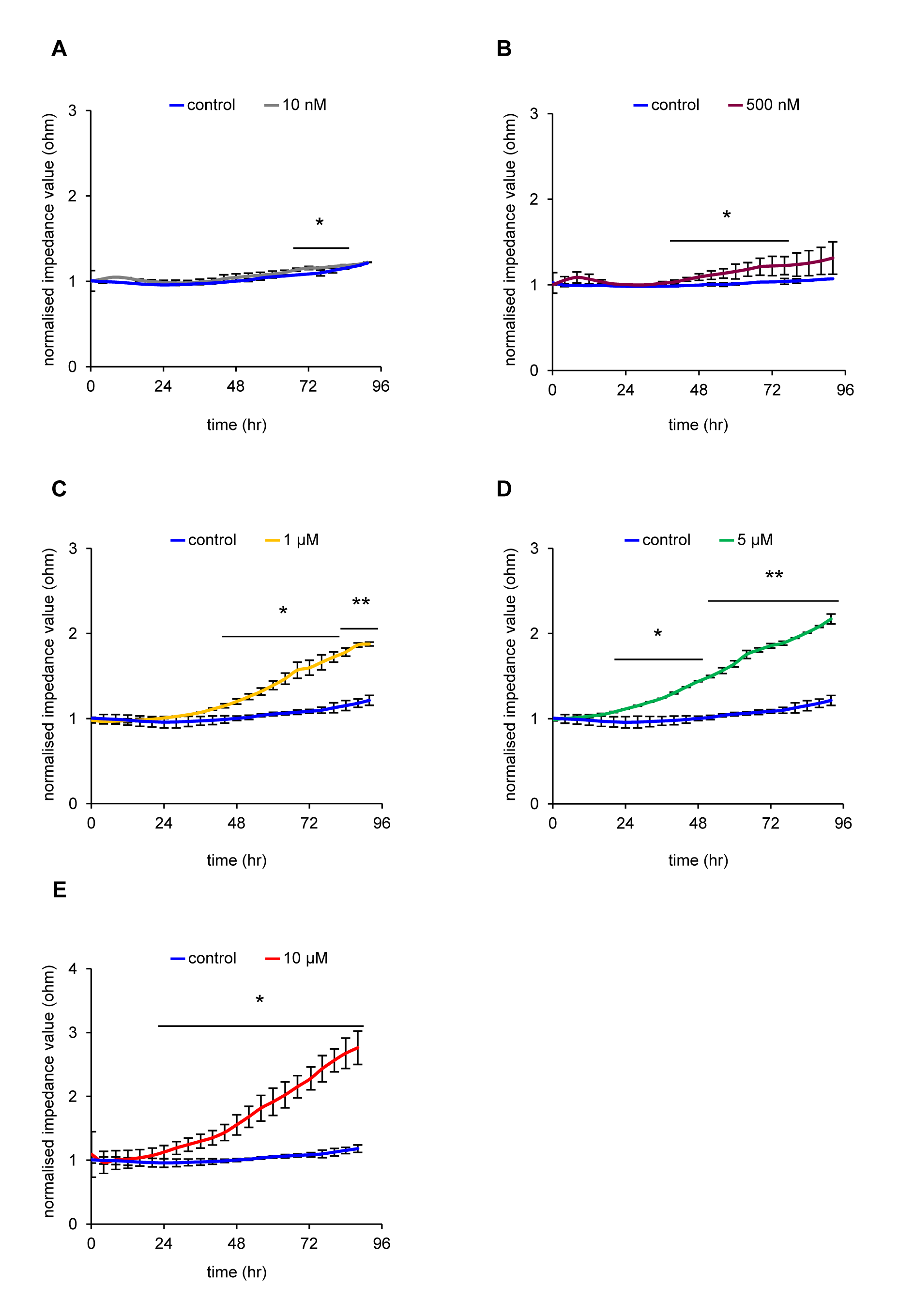

Supplement: Figure S1 — Induced concentration-dependent differentiation by RA. Impedance profiles comparing untreated NT2 with cells treated with 10 nM RA (A), 500 nM RA (B), 1 µM RA (C), 5 µM RA (D) and 10 µM RA (E) are shown. Measurements were executed at 45 kHz in 5-minute intervals for 96 hours. Each experiment was repeated at least three times. Standard deviations are indicated by error bars every four hours. Student’s t-test was used for statistical analysis (*p<0.05. **p<0.005). Black lines show regions with significant differences in respect to the untreated control. (TIF) [file pone.0059895.s001.tif]

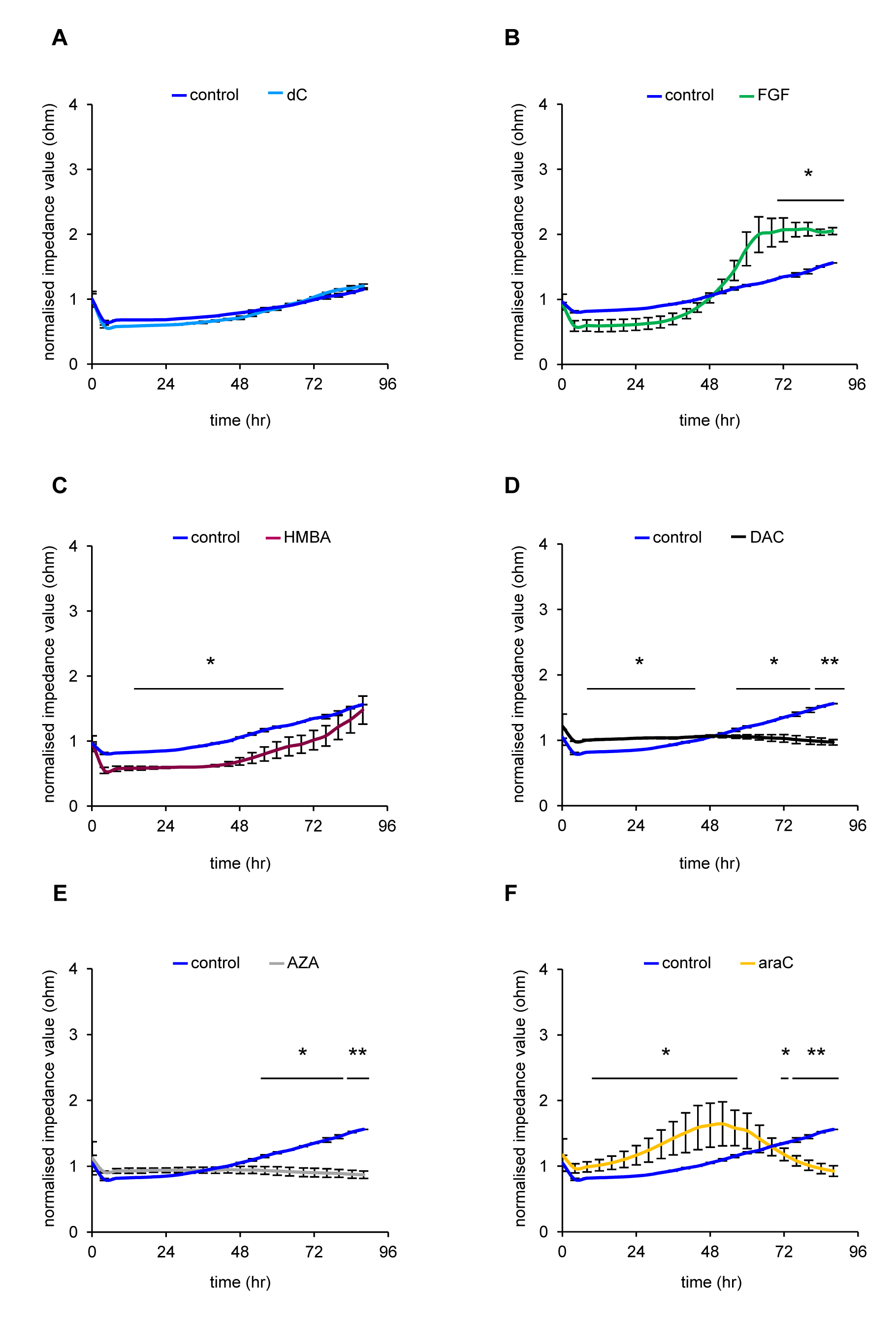

Supplement: Figure S2 — Induced differentiation by a panel of drugs. Impedance profiles comparing untreated NT2 with cells treated with 1 µM dC (A), 50 µM bFGF (B), 5 mM HMBA (C), 1 µM DAC (D), 1 µM AZA (E) and 1 µM araC (F) are shown. Measurements were executed at 45 kHz in 5-minute intervals for 96 hours. Each experiment was repeated at least three times. Standard deviations are indicated by error bars every four hours. Student’s t-test was used for statistical analysis (*p<0.05. **p<0.005). Black lines show regions with significant differences in respect to the dC control. (TIF) [file pone.0059895.s002.tif]

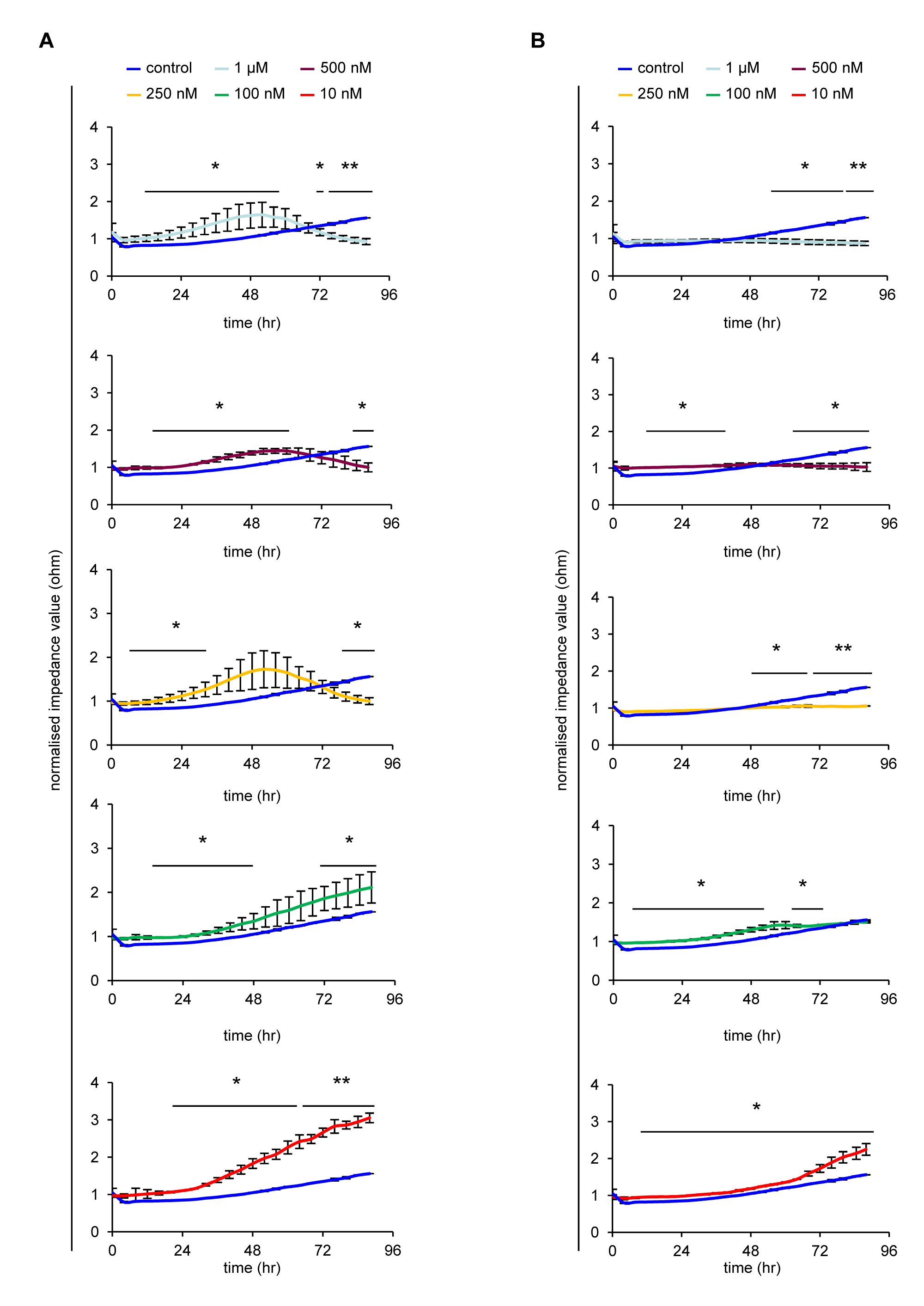

Supplement: Figure S3 — Induced concentration-dependent differentiation by araC and AZA. (A) Impedance profiles comparing untreated NT2 cells (dark blue) and cells treated with 1 µM (light blue), 500 nM (purple), 250 nM (yellow), 100 nM (green) and 10 nM (red) araC. (B) Impedance profiles comparing untreated NT2 cells (dark blue) and cells treated with 1 µM (light blue), 500 nM (purple), 250 nM (yellow), 100 nM (green) and 10 nM (red) AZA. Measurements were executed at 45 kHz in 5-minute intervals for 96 hours. Each experiment was repeated at least three times. Standard deviations are indicated by error bars every four hours. Student’s t-test was used for statistical analysis (*p<0.05. **p<0.005). Black lines show regions with significant differences in respect to the control. (TIF) [file pone.0059895.s003.tif]
