# Supplementary material for: Embryonic Carcinoma Cells Show Specific Dielectric Resistance Profiles during Induced Differentiation
Source: PLoS One. 2013 Mar 22;8(3):e59895. doi: 10.1371/journal.pone.0059895 (PMC3606267; doi:10.1371/journal.pone.0059895)
Supplement: Table S1 — Slope maxima of RA-treated NT2 cells. (PDF) [file pone.0059895.s004.pdf]

Table S1. Slope maxima of RA-treated NT2 cells

treatment	max. slope	time (h)	slope/time ratio
control	0.009055	91.59	0.000099
10 nM RA	0.005505	65.67	0.000094
500 nM RA	0.009422	68.72	0.000137
1 µM RA	0.020599	79.26	0.000260
5 µM RA	0.024929	59.42	0.000420
10 µM RA	0.029899	59.92	0.000499
