# Supplementary material for: Embryonic Carcinoma Cells Show Specific Dielectric Resistance Profiles during Induced Differentiation
Source: PLoS One. 2013 Mar 22;8(3):e59895. doi: 10.1371/journal.pone.0059895 (PMC3606267; doi:10.1371/journal.pone.0059895)
Supplement: Table S2 — Slope maxima of drug-treated NT2 cells. (PDF) [file pone.0059895.s005.pdf]

Table S2. Slope maxima of drug-treated NT2 cells

treatment	max. slope	time (h)	slope/time ratio
control	0.017534	100.76	0.000174
dC	0.015210	69.92	0.000218
RA	0.040009	75.84	0.000528
FGF	0.063303	56.26	0.001125
HMBA	0.052056	102.09	0.000510
DAC	0.006171	47.76	0.000129
AZA	0.004313	95.34	0.000045
araC	0.030684	40.67	0.000754
