# Supplementary material for: Embryonic Carcinoma Cells Show Specific Dielectric Resistance Profiles during Induced Differentiation
Source: PLoS One. 2013 Mar 22;8(3):e59895. doi: 10.1371/journal.pone.0059895 (PMC3606267; doi:10.1371/journal.pone.0059895)
Supplement: Table S3 — Slope maxima of araC- and AZA-treated NT2 cells. (PDF) [file pone.0059895.s006.pdf]

Table S3. Slope maxima of araC- and AZA-treated NT2 cells

treatment	max. slope	time (h)	slope/time ratio
control	0.016393	84.75	0.000193
10 nM araC	0.038219	59.58	0.000641
100 nM araC	0.040043	49.75	0.000805
250 nM araC	0.040542	40.25	0.001007
500 nM araC	0.042165	33.25	0.001268
1 µM araC	0.039774	33.92	0.001173
10 nM AZA	0.039077	72.08	0.000542
100 nM AZA	0.011582	43.17	0.000268
250 nM AZA	0.014941	46.25	0.000323
500 bM AZA	0.007572	37.08	0.000204
1 µM AZA	neg. slope	-	-
