# Supplementary material for: Embryonic Carcinoma Cells Show Specific Dielectric Resistance Profiles during Induced Differentiation
Source: PLoS One. 2013 Mar 22;8(3):e59895. doi: 10.1371/journal.pone.0059895 (PMC3606267; doi:10.1371/journal.pone.0059895)
Supplement: Table S4 — Slope maxima of OCT4-depleted NT2 cells. (PDF) [file pone.0059895.s007.pdf]

Table S4. Slope maxima of OCT4-depleted NT2 cells

treatment	max. slope	time (h)	slope/time ratio
control	0.012303	142.67	0.000086
kd OCT4	0.037599	92.67	0.000406
