# Supplementary material for: Embryonic Carcinoma Cells Show Specific Dielectric Resistance Profiles during Induced Differentiation
Source: PLoS One. 2013 Mar 22;8(3):e59895. doi: 10.1371/journal.pone.0059895 (PMC3606267; doi:10.1371/journal.pone.0059895)
Supplement: Table S5 — RT-Primer pairs used in this study. (PDF) [file pone.0059895.s008.pdf]

Table S5. RT-Primer pairs (5´-3´):

HOXA1	for<br>rev	GCCGTACTCTCCAACTTTC<br>CTCGCCTCAATACATTCACC
SNAP25	for<br>rev	GCCAGATCGACAGGATCATG<br>CAACCTTTGGAAAGAAAGAGAC
NANOG	for<br>rev	CCTCAGCCTCCAGCAGATG<br>GGTCTGGTTGCTCCACATTG
OCT4	for<br>rev	CTGACAACAATGAAAATCTTCAG<br>GTTACAGAACCACACTCGGAC
ß-ACTINE	for<br>rev	GATCAAGATCATTGCTCCTCCTG<br>CTAGAAGCATTTGCGGTGGAC
NESTIN	for<br>rev	GCGGCTGCGGGCTACTGAAA<br>CCAGGAGGGTCCTGTACGTGGC
ß-TUBULIN III	for<br>rev	CACGAGCACACTGTGGGGCA<br>ACTTCCCAGAACTGTGGACGCCTG
